# Supplementary material for: Predicting visual function by interpreting a neuronal wiring diagram
Source: Nature. 2024 Oct 2;634(8032):113–23. doi: 10.1038/s41586-024-07953-5 (PMC11446822; doi:10.1038/s41586-024-07953-5)
Supplement: Supplementary file 6 — CRF and ERF predictions for individual TmY4 and TmY9 cells. Analogous to Supplementary Data 3, but for TmY target types. Shown are the top four monosynaptic pathways, the strongest pathway passing through each of the top ten intermediary types (ranking from Extended Data Fig. 7), and the trisynaptic pathway Tm1–TmY–Dm3–TmY (see the section entitled Prediction of spatial normalization). [file 41586_2024_7953_MOESM6_ESM.zip › DataS4/TmY9qΓèÑ/720575940641552117.pdf]

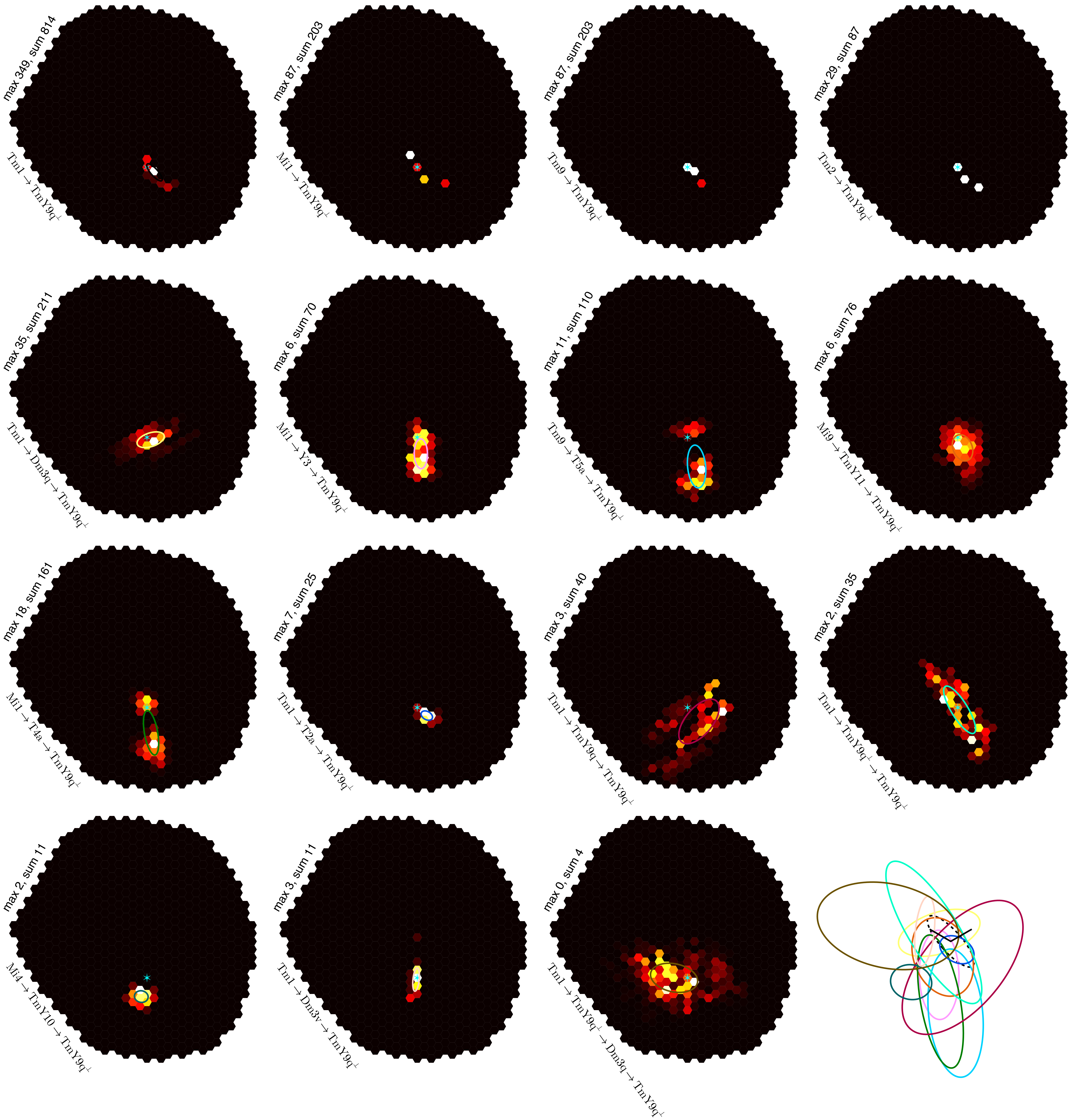
max 349, sum 814
Tm1 → TmY9q⊥
max 87, sum 203
Mi1 → TmY9q⊥
max 87, sum 203
Tm9 → TmY9q⊥
max 29, sum 87
Tm2 → TmY9q⊥
max 35, sum 211
Tm1 → Dm3q → TmY9q⊥
max 6, sum 70
Mi1 → Y3 → TmY9q⊥
max 11, sum 110
Tm9 → T5a → TmY9q⊥
max 6, sum 76
Mi9 → TmY11 → TmY9q⊥
max 18, sum 161
Mi1 → T4a → TmY9q⊥
max 7, sum 25
Tm1 → T2a → TmY9q⊥
max 3, sum 40
Tm1 → TmY9q⊥ → TmY9q⊥
max 2, sum 35
Tm1 → TmY9q⊥ → TmY9q⊥
max 2, sum 11
Mi4 → TmY10 → TmY9q⊥
max 3, sum 11
Tm1 → Dm3v → TmY9q⊥
max 0, sum 4
Tm1 → TmY9q⊥ → Dm3q → TmY9q⊥
